# The Importance of Exercising Caution When Comparing Results from Malaria Vaccines Administered on the EPI Schedule and on a Seasonal Schedule

**DOI:** 10.4269/ajtmh.22-0544a

**Published:** 2022-11-21

**Authors:** Ashley Birkett, R. Scott Miller, Lorraine A. Soisson

**Affiliations:** PATH Center for Vaccine Innovation and Access Seattle, Washington E-mail: abirkett@path.org; Bill & Melinda Gates Medical Research Institute, Malaria Program Cambridge, Massachusetts E-mail: scott62miller@outlook.com; CAMRIS International, LLS, a USAID Contractor, USAID Malaria Vaccine Development Program, Washington, District of Columbia E-mail: soisson88@gmail.com

Dear Sir,

We are writing in response to Phil Rosenthal’s article “Malaria in 2022: Challenges and Progress,” which was published recently in the *American Journal of Tropical Medicine and Hygiene*.^1^ In that article, Dr. Rosenthal states that “new data on the R21 vaccine, which is also based on CSP [circumsporozoite protein] antigens, with preventive efficacy ∼75% in African children, [was] considerably greater than that seen in RTS,S trials.” Although the data published by the University of Oxford and partners on the Phase 2 results of their trial in Burkina Faso did demonstrate high levels of efficacy,^2^ the data cannot be reliably compared with Phase 3 trial results for RTS,S/AS01 (RTS,S).

The R21/Matrix-M (R21) Phase 2 trial was conducted at one center in one country only. The study reported vaccine efficacy of 77% (95% CI: 66–84) in Nanoro, Burkina Faso, over 12-months of follow-up.^2^ The study was performed in a highly seasonal transmission setting, with vaccination coordinated to ensure peak immunity at the time of peak risk (so-called seasonal vaccination); peak risk occurred during the first 6 months of follow-up, followed by the low transmission dry season, with only a single clinical case observed in the control arm between days 200 and 300 of follow-up. It is therefore most appropriate to express these data as efficacy over one season and/or 6 months to align with the period of risk. Additional data, in settings of perennial transmission or over multiple seasons in regions of highly seasonal transmission, are needed to determine appropriately the efficacy of this vaccine candidate over longer follow-up periods and to enable more meaningful comparison to the RTS,S Phase 3 data.

The RTS,S Phase 3 trial was conducted at 11 centers across seven countries, including regions of both seasonal and perennial transmission. Aligned with the EPI schedule for dosing based on child age, the RTS,S vaccine delivery was not optimized to achieve peak immunity during the high transmission setting, nor do 6-month data occur during highest force of infection.^3^ The vaccine efficacy against clinical malaria, over 6 months of follow-up, across all 11 sites, was 68% (95% CI: 64–72); at the same Nanoro site, the vaccine efficacy was 72% (95% CI: 60–80).^4^ Direct comparisons of relatively short-acting vaccines or monoclonal antibodies employing different delivery strategies should be avoided, and hence the data available to date do not support the superiority of one vaccine over another.

We look forward to learning more about the longer-term follow-up from the promising R21 Phase 2 trial and from the ongoing Phase 3 trial, which will provide more definitive information about the vaccine candidate’s efficacy, durability, and safety in a significantly larger population and across multiple malaria transmission settings. The availability of additional malaria vaccines, complementing RTS,S, could be highly beneficial to comprehensive malaria control efforts.
